# Non-nutritive Sweeteners and Health: Reconciling Evidence and Interrogating Guideline Disconnects

**DOI:** 10.1016/j.advnut.2024.100328

**Published:** 2024-12-02

**Authors:** Tauseef A Khan, Sabrina Ayoub-Charette, John L Sievenpiper

**Affiliations:** 1 Department of Nutritional Sciences, Faculty of Medicine, University of Toronto, Toronto, Ontario, Canada; 2 Toronto 3D Knowledge Synthesis and Clinical Trials Unit, Clinical Nutrition and Risk Factor Modification Centre, St. Michael's Hospital, Toronto, Ontario, Canada; 3 Division of Endocrinology and Metabolism, Department of Medicine, St. Michael's Hospital, Toronto, Ontario, Canada; 4 Li Ka Shing Knowledge Institute, St. Michael’s Hospital, Toronto, Ontario, Canada; 5 Department of Medicine, Faculty of Medicine, University of Toronto, Toronto, Ontario, Canada

Recent dietary and clinical practice guidelines endorse non-nutritive sweeteners (NNSs) as a viable strategy for reducing calories from added/free sugars and achieving weight loss benefits [1,2]. However, the WHO’s recent guideline advises against using NNS for weight control or reducing noncommunicable disease risks for both adults and children [3]. This recommendation has sparked significant debate within the scientific community [4], as it appears to conflict with other nutrition and clinical practice guidelines [2].

The WHO guideline is based upon a commissioned systematic review and meta-analysis (SRMA) of randomized controlled trials (RCTs) and prospective cohort studies, where RCTs consistently showed benefit of NNS in substitution for added/free sugars for weight loss and calorie deficit [5]. However, prospective cohort studies, which used prevalent analysis, showed increased association with obesity and chronic disease outcomes such as cardiovascular disease and stroke, and total mortality. Over the past decade, numerous SRMAs on NNSs and their impacts on body weight and other cardiometabolic outcomes have been published. The majority echo the findings of the WHO-commissioned SRMA, highlighting contrasting evidence between RCTs and prospective cohort studies.

In this issue, Higgins et al. [6] provide an overview of these SRMAs, focusing on the relationship between NNS consumption and body weight. Analyzing 20 SRMAs of RCTs (*n* = 75), prospective cohort studies (*n* = 42), and cross-sectional studies (*n* = 10), the authors underscore methodologic differences that lead to varying conclusions. Despite these differences, the authors show that meta-analyses of RCTs consistently demonstrate that NNS are beneficial for weight management, particularly when compared with sugars. This suggests that NNS effectively displace calories from sugars. Conversely, SRMAs of prospective cohort studies, which, all except for one, used prevalent analyses, present mixed results in largely the opposite direction. Prevalent analyses, which measure NNS use at a single point in time (usually at baseline) as a measure of usual intake, frequently show no association or an increase in body weight. This method is at high risk of reverse causality, as individuals with existing health risks may be using NNS as a strategy to mitigate that risk by managing calories and sugars intake [7]. The one SRMA of cohort studies, which did not show signals of harm, was commissioned by the Diabetes and Nutrition Study Group for the update of the European Association of Diabetes (EASD) clinical practice guidelines for nutrition therapy. It was specifically designed to mitigate the issues of reverse causality and residual confounding by adjustments for adiposity and examining the substitution NNS for sugars, in the form of replacing sugar-sweetened beverages (SSBs) with NNS beverages, and reported that this substitution was associated with lower body weight [7].

There has been particular concern about the use of NNS in children. As sugar reduction strategies gain traction, NNS consumption among children and adolescents has surged globally sparking concerns of overconsumption of NNS in this group [8]. Current evidence from RCTs indicates that NNS, within acceptable daily intake limits, are not associated with harm in children but rather benefit in reducing calories and body weight [5,9]. Surveys show that even in high-NNS-intake countries like Chile, children do not exceed acceptable daily intake limits [10]. As the latest WHO guidance on NNS recommends against NNS for sugar reduction in children, there is a pressing need to update the evidence base in this age group.

Espinosa et al. [11] in this issue address this pressing need with an SRMA focused solely on NNS intake and BMI in children and adolescents. Analyzing four RCTs and eight cohort studies, they found that NNS beverages reduced BMI compared with SSBs in RCTs, especially in populations with obesity and long-term interventions (>34 wk). This finding suggests that NNS benefits stem from displacing calories from sugars. Conversely, prospective cohort studies found no association between NNS beverages and BMI changes, even in studies using change analysis. One reason the latter did not show benefit might be that the calorie displacement did not have enough time to show an association or the number of studies were limited. There was insufficient data to allow for substitution analyses. The authors acknowledge limitations of observational studies and methodologic concerns, such as relying on single dietary estimates to predict BMI changes.

The two studies highlight persistent issues in NNS research and underscore the need for rigorous evidence synthesis methods to resolve the role of NNS in replacing sugars and excess calories. The confusion created by WHO guidance must be addressed with high-quality, robust analyses that better protect against reverse causality and residual confounding. RCTs, the gold standard for establishing causality, provide strong and consistent evidence supporting the benefits of NNS in substitution for sugars in weight management, which is shown by both Higgins et al. [6] and Espinosa et al. [11]. The WHO guideline, however, discounted the higher certainty evidence from RCTs and based their recommendation on prospective cohort studies [4,12]. Although prospective cohort studies provide the best protection against bias among observational studies, this level of evidence does not allow for causal inferences, ranking below RCTs in the hierarchy of evidence [13]. This issue is compounded by reliance on prevalent exposure analyses in most syntheses of prospective cohort studies, including the WHO-commissioned synthesis and resulting guideline [4]. The WHO guideline overlooked recent advances in analytical methods development that mitigate these important sources of bias. Techniques like change and substitution analyses, which account for changes in NNS over time and substitution of NNS for sugars, better model and align with RCT findings, supporting the role of NNS in calorie reduction and weight management [4,7]. Relying on evidence at high risk of bias from prevalent analyses of prospective cohort studies over more robust evidence from RCTs and change and substitution analyses of prospective cohort studies undermines guidance.

To ensure that NNS research produces consistent and high-quality results, the focus should be on enhancing rigor in the field through a 3-pronged approach. First, future observational studies and their synthesis should use rigorous methods, such as change and substitution analyses with adjustment for adiposity, to reduce bias from reverse causality and residual confounding. By using such methods, researchers can obtain more accurate estimates of the relationship between NNS and cardiometabolic health. Second, high-quality long-term RCTs are indispensable. These RCTs should transition from idealized settings to investigate commonly consumed NNS-containing foods and beverages in real-world contexts, using both caloric and noncaloric comparators. Such pragmatic trials would better reflect consumer behavior and provide robust evidence to inform future systematic reviews, meta-analyses, and ultimately, public health guidelines. Finally, the field should bridge the gap between mechanistic studies, animal models, controlled trials, and observational research by aligning these bodies of evidence [14]. This can be achieved by improving clarity of causal questions and refining study designs using robust methodologic advancements at every level. Current discrepancies in methodologies, such as the use of non-translatable doses and blends hinder progress and limit the applicability of mechanistic findings to humans. Mechanistic studies on NNS, particularly those investigating acute metabolic outcomes and gut microbiota, should prioritize doses, combinations, and comparators that accurately reflect typical consumer exposure. This will ensure the translation of insights from pre-clinical studies to populations under real-world conditions.

In conclusion, discrepancies in NNS results between RCTs and prospective cohorts are primarily due to methodologic limitations. Increasing methodologic rigor in study design is essential to demonstrate accurately the role of NNS in reducing caloric intake. Guidelines based on rigorous methods yield clear and consistent recommendations, as seen in the latest EASD guideline [2], although those lacking rigor, like the WHO guideline, can be ambiguous [3]. Moving forward, it is incumbent on investigators to produce high-quality data, guidelines, and policymakers to base guidelines and policy on the best evidence using the most robust synthesis methods, and users to demand the highest level of rigor in guidelines and policy development to ensure we have both clarity and reliability in this controversial field.

## Author contributions

TAK, SAC: wrote the first draft; JLS: revised and updated the draft; and all authors: contributed equally to the manuscript and have read and approved the final version.

## Conflict of interest

TAK reports receiving grants from Institute for the Advancement of Food and Nutrition Sciences (Advancement of Food and Nutrition Sciences (IAFNS), formerly International Life Sciences Institute (ILSI) North America), and National Honey Board (U.S. Department of Agriculture (USDA) Checkoff program). He has received honorariums from IAFNS, the International Food Information Council (IFIC), and the Calorie Control Council (CCC), the International Sweeteners Association (ISA), and AmCham Dubai. He has received funding from the Toronto 3D Knowledge Synthesis and Clinical Trials foundation. JLS has received research support from the Canadian Foundation for Innovation, Ontario Research Fund, Province of Ontario Ministry of Research and Innovation and Science, Canadian Institutes of health Research (CIHR), Diabetes Canada, American Society for Nutrition (ASN), National Honey Board (USDA honey “Checkoff” program), Institute for the IAFNS, Pulse Canada, Quaker Oats Center of Excellence, INC International Nut and Dried Fruit Council Foundation, The United Soybean Board (USDA soy “Checkoff” program), Protein Industries Canada (a Government of Canada Global Innovation Cluster), Almond Board of California, European Fruit Juice Association, The Tate and Lyle Nutritional Research Fund at the University of Toronto, The Glycemic Control and Cardiovascular Disease in Type 2 Diabetes Fund at the University of Toronto (a fund established by the Alberta Pulse Growers), The Plant Protein Fund at the University of Toronto (a fund which has received contributions from International Flavors & Fragrances Inc. (IFF) among other donors), The Plant Milk Fund at the University of Toronto (a fund established by the Karuna Foundation through Vegan Grants), and The Nutrition Trialists Network Fund at the University of Toronto (a fund established by donations from the Calorie Control Council, Physicians Committee for Responsible Medicine and Login5 Foundation). He has received food donations to support randomized controlled trials from the Almond Board of California, California Walnut Commission, Danone, Nutrartis, Soylent, and Dairy Farmers of Canada. He has received travel support, speaker fees, and/or honoraria from FoodMinds LLC, Nestlé, Abbott, General Mills, Nutrition Communications, IFIC, Arab Beverage Association, International Sweeteners Association, Calorie Control Council, and Phynova. He has or has had ad hoc consulting arrangements with Almond Board of California, Perkins Coie LLP, Tate & Lyle, Ingredion, and Brightseed. He is on the Clinical Practice Guidelines Expert Committees of Diabetes Canada, EASD, Canadian Cardiovascular Society (CCS), and Obesity Canada/Canadian Association of Bariatric Physicians and Surgeons. He serves as an unpaid member of the Board of Trustees of IAFNS. He is a Director at Large of the Canadian Nutrition Society (CNS), founding member of the International Carbohydrate Quality Consortium (ICQC), Executive Board Member of the Diabetes and Nutrition Study Group (DNSG) of the EASD, and Director of the Toronto 3D Knowledge Synthesis and Clinical Trials foundation. His spouse is a former employee of Nestle Health Science and AB InBev. SAC has no conflicts to declare.

## Funding

SAC is funded by Canadian Institute of Health Research (CIHR)-Doctoral award. JLS was funded by Diabetes Canada Clinician Scientist award.
